# Risk factors for moderate-to-severe postoperative pain after percutaneous nephrolithotomy: a retrospective cohort study

**DOI:** 10.1038/s41598-022-12623-5

**Published:** 2022-05-19

**Authors:** Haotian Wu, Tianfu Ding, Siyi Yan, Zhongyue Huang, Huan Zhang

**Affiliations:** 1 School of Clinical Medicine, Tsinghua University, Beijing, China; 2 Department of Anesthesiology, Beijing Tsinghua Changgung Hospital, No.168 Litang Road, Changping District, Beijing, 102218 China

**Keywords:** Risk factors, Urology

## Abstract

Percutaneous nephrolithotomy (PCNL) is a minimally invasive procedure for removing renal calculi, while a large number of patients experience acute moderate-to-severe pain despite the analgesia provided. This study aimed to explore the risk factors for postoperative pain after PCNL, which may provide a novel perspective to refine the enhanced recovery after surgery (ERAS) program and to improve clinical outcomes. The clinical data of 331 patients who underwent PCNL in our hospital from September 2020 to February 2021 were retrospectively analyzed. The pain intensity was assessed every 4 h until 24 h post-surgery. According to the visual analog scale (VAS) score, patients were divided into two groups: mild or no pain group (VAS score, 0–3) and moderate-to-severe pain group (VAS score, 4–10). The pre-, peri-, and post-operative data were collected and analyzed. The indicators with statistically significant differences were selected, and multivariate logistic regression analysis was employed to determine the risk factors for postoperative pain after PCNL. Among 331 patients, 221 patients had moderate-to-severe pain and the incidence rate was 66.77%. Multivariate logistic regression analysis showed that the independent risk factors for moderate-to-severe pain after PCNL were the diameter of the renal calculus (odds ratio (OR) = 6.23, 95% confidence interval (CI) 2.50–15.56, P = 0.001), the number of renal calculi (OR = 15.892, 95% CI 7.721–32.711, P < 0.01), the presence of residual calculi (OR = 1.780, 95% CI 0.897–3.533, P = 0.01), and operation time (OR = 1.033, 95% CI 1.020–1.046, P < 0.01). The diameter of the renal calculus, the number of renal calculi, the presence of residual calculi, and operation time were significant predictors of postoperative pain after PCNL.

## Introduction

Over the past two decades, open surgery has been almost completely replaced by minimally invasive procedures for patients with kidney stones^1^. According to the European Association of Urology guidelines, percutaneous nephrolithotomy (PCNL) is the first-line treatment for large (> 2 cm), multiple, and inferior calyx renal stones^2^.

However, to date, few studies have concentrated on the postoperative pain after PCNL. Minimally invasive surgery is not painless, and postoperative pain after PCNL includes creation of a percutaneous access tract through the parenchyma and parenchymal shearing, renal pelvic pressure, renal tissues, visceral pain mainly accompanied by autonomic nerve reaction, and low back pain caused by indwelling nephrostomy tube^3–5^. It may originate from the renal capsule, muscle, subcutaneous tissues, and skin^6^.

Anatomical studies showed that the main sources of acute postoperative pain after PCNL were visceral pain from kidneys and ureters and body surface pain from incisions. Renal pain originates from the T10-L1 spinal nerve, and ureteral pain originates from the T10-L2 spinal nerve. However, the incisions and pathways are typically formed under the 12th rib or between the 10th and 11th ribs, where a cutaneous innervation is mainly undertaken by T10–11. Indwelling of renal fistula, peritubular compression of renal cortex, and dilation of renal capsule may also aggravate postoperative pain. However, discomfort, stress, and pain associated with nephrostomy highlight the importance of postoperative analgesia. Additionally, stones were discharged from the ureter due to changes in body position when residual stones or large stones (4–5 mm) were directly flushed into the ureter due to fluid flow intraoperatively and moved into the ureter. Because calculi stimulate mucous membrane of the ureter and cause ureteral spasm,ache typically lasts longer^7^. Since the urinary system is innervated by the same autonomic nerve as the gastrointestinal system, pain may cause reflexive nausea and vomiting^4^.

The pain is mainly associated with severe postoperative somatosensory and visceral pain, necessitating a substantial action for postoperative opioid consumption^8^. If postoperative pain is not timely alleviated, it may result in adverse consequences, such as delay of activity, pulmonary dysfunction, prolonged hospitalization, and even septic shock and renal failure in some severe cases^9^. More importantly, transition of acute pain to chronic pain may occur, seriously influencing patients’ quality of life.

The present study aimed to identify the predictors for the postoperative pain after PCNL, which may provide a novel perspective to refine the enhanced recovery after surgery (ERAS) program and to improve clinical outcomes.

## Materials and methods

### Study design and patients

The study protocol was approved by the Ethics Committee of Beijing Tsinghua Changgung Hospital (Beijing, China; Approval No. 21452-4-02), and the study was conducted in accordance with the tenets of the Declaration of Helsinki. Written informed consent was obtained from all eligible subjects or their entrusted representatives before enrollment.

The clinical data of 331 patients who underwent PCNL in Beijing Tsinghua Changgung Hospital from September 2020 to February 2021 were retrospectively analyzed. In conformity with the occurrence of postoperative pain, patients were divided into mild or no pain group and moderate-to-severe pain group. The inclusion criteria were as follows: (1) patients undergoing elective PCNL under general anesthesia; (2) patients aged 18–70 years old; (3) American Society of Anesthesiologists (ASA) grade I–III; (4) body mass index (BMI) of 18–35 kg/m^2^; and (5) signing the written informed consent form. The exclusion criteria were as follows: (1) history of surgery for ipsilateral renal stones; (2) severe urinary tract infection; (3) severe cardiac and pulmonary insufficiency and coagulation disorders; (4) a spinal deformity, an underlying pathology, or history of surgery for prior spinal cord injury; (5) neurological disorders, psychiatric disorders or experience of substance abuse.

According to the ordinary preoperative fasting guidelines, patients were prohibited from both food and fluids for at least 8 h prior to surgery. A preventive antibiotic (ertapenem, 1000 mg) was given 30 min before surgery. Venous access was established in the upper extremities, and the blood pressure (BP), electrocardiogram (ECG), heart rate (HR), and pulse oxygen saturation (SpO_2_) were routinely monitored.

### Anesthetic and surgical procedures

All patients underwent general anesthesia using the same type of medication. General anesthesia was induced with intravenous propofol (2 mg/kg) plus sufentanil (0.4 µg/kg), and neuromuscular blockade was achieved with rocuronium (0.6 mg/kg) and maintained with combined intravenous–inhalation anesthesia. After endotracheal intubation, the mechanical ventilation was performed with tidal volume of 6–8 mL/kg, 12 breaths/min. Anesthesia was maintained with a minimum alveolar concentration (MAC) of sevoflurane (1.5–2.5%) in a 60% oxygen and air. Nitrous oxide was not used. Remifentanil infusions (0.1–0.2 μg/kg/min) were used for analgesia. Expiratory CO_2_, SpO_2_, HR, and BP were monitored continuously to ensure the maintenance of anesthesia. The MAP was maintained at 60–100 mmHg, the bispectral index (BIS) at 40–60%, and these parameters were measured every 5 min throughout the procedures. After proper padding of eyes, patients were kept in prone position, and all pressure points were secured.

#### Puncture

**Figure 1:**
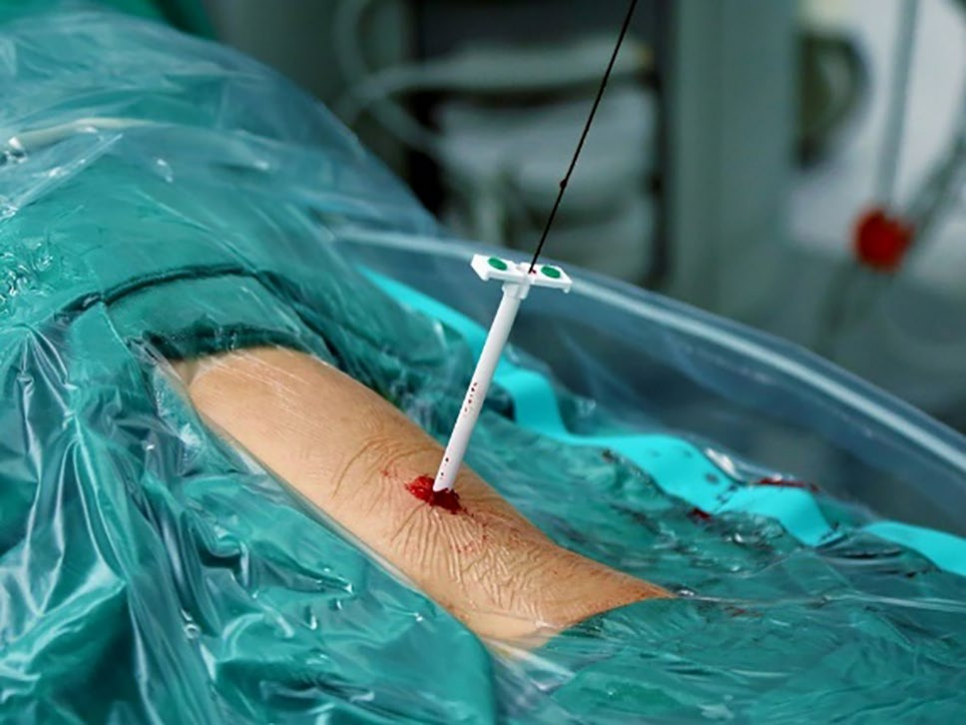
Under the guidance of ultrasound, the puncture needle selected the puncture point between the 11th intercostal or 12th intercostal posterior axillary line and the subscapular angle to puncture into the target calyces and establish the 16F microchannel. (taken by Tianfu Ding).

**Figure 2:**
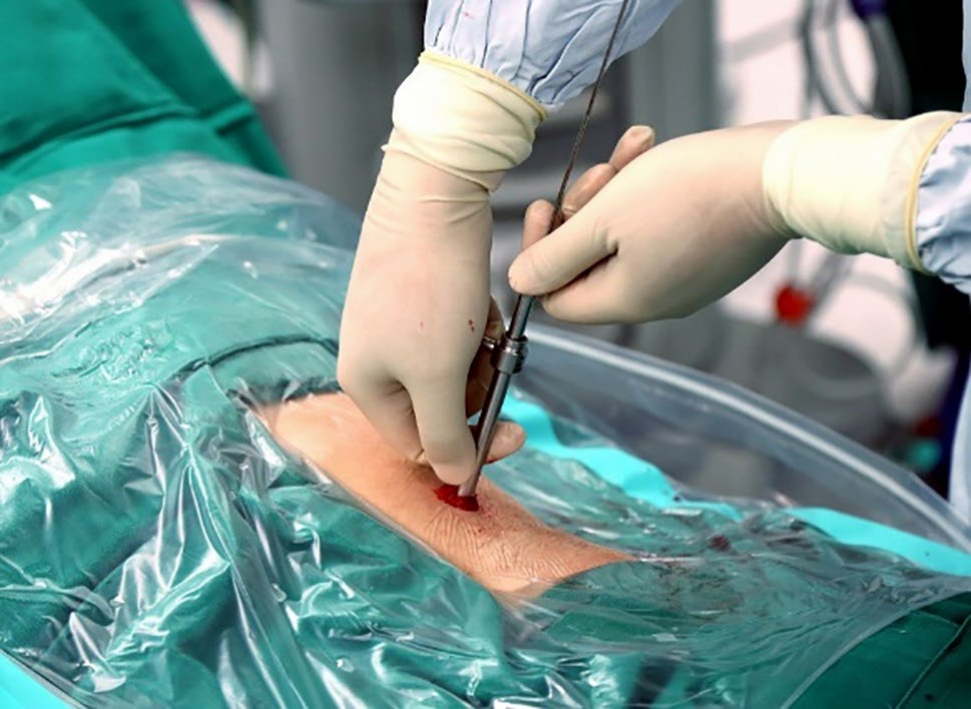
The "two-step" method was used to establish the standard channel. First, a small ureteroscope was used to enter the microchannel to observe the position of the channel, and the preliminary adjustment was made. After the adjustment, the sheath was gradually expanded to establish the 24F standard channel. (taken by Tianfu Ding).

Under the guidance of B-mode ultrasound, the area between the posterior axillary line and the scapular line was selected as the puncture point under 12 ribs or between 11 ribs according to the distribution of calculi. After puncturing the target calyx, a guide wire was placed to dilate. Two-step or balloon dilation can be used to establish a standard channel (Fig. 1). In the present study, pneumatic ballistic lithotripsy was selected. Different stones could be removed with gravel forceps, hanging baskets, and other instruments (Fig. 2). Stones were broken into small pieces and then sucked out or washed with a detergent. Larger stones could be removed with stone forceps, and B-mode ultrasound was used to observe postoperative residual stones. Nephrostomy tube was placed at 3 days after surgery, and the extubation time was appropriately advanced or prolonged according to the intraoperative and postoperative recovery. Ureteral stents should be retained for 2–4 weeks after surgery. If ureteral stenosis occurs, the duration of the use of ureteral stents should be extended^10^.

At 30 min before the end of surgery, 100 mg of flurbiprofen axetil and 8 mg of ondansetron were administered intravenously. After surgery, patients were transferred to post-anesthesia care unit (PACU) for recovery. Neostigmine and tropine were used to antagonize residual neuromuscular block when necessary. Endotracheal tube was removed if patients were awake with absence of respiratory failure, residual paralysis, and overdose of opioids. All patients were extubated in PACU, and were then sent to the ward when their modified Aldrete score was more than 13.

### Evaluation of pain

Participants’ pain was assessed using the visual analog scale (VAS) scoring system. It is a standard and verified 10-point scale for pain self-report, where a score of 0 represents no pain and a score of 10 represents the highest pain level. VAS scores were assessed by a well-trained doctor at 30 min immediately after extubation, followed by every other 4 h until 24 h post-surgery in the ward.

### Routine analgesia protocol and rescue analgesia

All patients did not receive patient-controlled intravenous analgesia (PCIA) postoperatively. The pain intensity was assessed using the VAS scoring system during rest and cough every 4 h thereafter until 24 h post-surgery. Patients were divided into two groups based on VAS pain score: mild or no pain (VAS score, 0–3) and moderate-to-severe pain (VAS score, 4–10). Rescue analgesia was intravenously administered according to the protocol, and patients were treated with parecoxib sodium (40 mg IV) when they complained of moderate-to-severe pain and two injections should be given at least four hours apart.

Data collection was performed by urologists and anesthesiologists for age, gender, BMI, ASA grade, diameter of the renal calculus, degree of hydronephrosis, preoperative urine culture, expansion method, tract diameter, the presence of residual calculi, preoperative analgesia, underlying disease, puncture time, number of expanded channels, operation time, postoperative complications (Clavien–Dindo classification), number of renal calculi, and VAS pain score. Postoperative adverse events included postoperative nausea and vomiting (PONV), high and low BP, bradycardia, pruritus, and respiratory depression.

### Statistical analysis

SPSS 25.0 software (IBM, Armonk, NY, USA) was used to perform statistical analysis. Measurement data were expressed as mean ± standard deviation and were analyzed by the *t*-test or χ^2^ test. The Kolmogorov-Smirnoff single-sample test was used to assess the normal distribution of continuous variables before further comparisons. The Chi-square test was utilized for comparison of the two groups. When *P* was < 0.05, the difference was regarded as statistically significant. Multivariate logistic regression analysis was used to analyze the statistical significance of the indicators.

### Ethics approval and consent to participate

This study was approved by the Ethics Committee of Beijing Tsinghua Changgung Hospital. All patients signed the informed consent form. The procedures did not require any additional intervention, and all data were analyzed anonymously. All methods were carried out in accordance with relevant guidelines and regulations.

## Results

In the present study, 331 patients from a single center were enrolled. Among them, 221 patients had moderate-to-severe pain, with an incidence of 66.77%. The results showed that the number of renal calculi (*P* < 0.01), the diameter of the renal calculus (*P* < 0.01), the presence of residual calculi (*P* = 0.017), and operation time (*P* < 0.01) were significantly different between the two groups (Table 1). Multivariate logistic regression analysis showed that the independent risk factors for postoperative pain after PCNL included the diameter of the renal calculus (odds ratio (OR) = 6.23, 95% confidence interval (CI) 2.50–15.56, P = 0.001), the number of renal calculi (OR = 15.892, 95% CI 7.721–32.711, P < 0.01), the presence of residual calculi (OR = 1.780, 95% CI 0.897–3.533, P = 0.01), and operation time (OR = 1.033, 95% CI 1.020–1.046, P < 0.01) (Table 2).

**Table 1:** Comparison of postoperative pain factors between the two groups.

	Mild or no pain group (n = 110) (33.3)	Moderate-to-severe pain group (n = 221) (66.7)	*t/F*	*p*
	Median 2	Median 7		
**Gender (n)**			0.163	0.687
Female	39 (32)	83 (68)		
Male	71 (34.1)	137 (65.9)		
Age (years, mean ± SD)	51.24 ± 11.10	50.72 ± 11.98		
**BMI (kg/m**^**y**^**)**			0.00	1.00
≤ 25	51 (33.3)	102 (66.7)		
> 25	59 (33.3)	118 (66.7)		
**ASA classification**			2.72	0.099
I	37 (40.2)	55 (59.8)		
II	73 (30.7)	165 (69.3)		
Renal calculusdiameter (mm, mean ± sd )	27.07 ± 13.86	31.95 ± 16.06	− 2.72	< 0.01
**Degree of hydronephrosis**			4.014	0.247^a^
Normal	57 (36.5)	99 (63.5)		
Mild	46 (30.3)	106 (69.7)		
Moderate	2 (16.7)	10 (83.3)		
Severe	5 (50)	5 (50)		
**Preoperative urine culture**			0.66	0.417
Negative	74 (34.9)	138 (65.1)		
Positive	36 (30.5)	82 (69.5)		
**Expansion method**			1.778	0.182
Balloon dilatation	54 (37.2)	91 (62.8)		
Two-step method Dilatation	56 (30.3)	129 (69.7)		
**Tract diameter (Fr)**			2.056	0.152
24Fr	109 (34)	212 (66)		
< 24Fr	1 (11.1)	8 (88.9)		
**Calculusresidual**			5.72	0.017
Calculus-free	87 (37.3)	146 (62.7)		
Calculus-residual	23 (23.7)	74 (76.3)		
**Preexistent medication**			0.228	0.892
None	95 (33.8)	186 (66.2)		
Nonsteroidal	12 (30)	28 (70)		
Weak opioids	3 (33.3)	6 (66.6)		
**Comorbidities**			1.81	0.77
None	68 (31.3)	149 (68.7)		
Hypertension	22 (40.7)	32 (59.3)		
Diabetes	13 (35.1)	24 (64.9)		
coronary heart disease	5 (31.3)	11 (68.8)		
Hyperlipidemia	2 (33.3)	4 (66.7)		
Puncture time (s, mean ± sd)	206.80 ± 43.69	213.66 ± 39.51	− 1.44	0.152
**Number of expanded channels (n)**			3.357	0.187
1	98 (35.4)	179 (64.6)		
2	11 (23.4)	36 (76.6)		
3	1 (16.7)	5 (83.3)		
Duration of surgery (min, mean ± SD)	85.07 ± 27.22	110.93 ± 38.14	− 6.35	< 0.01
**Postoperative complications**			4.207	0.120
Normal	99 (32.7)	204 (67.3)		
Clavien–Dindo I^b^	11 (47.8)	12 (52.2)		
Clavien–Dindo II	0 (0)	4 (100)		
Preoperative creatinine (µmol/L, mean ± SD)	85.11 ± 50.32	79.22 ± 45.01	1.078	0.282
**Number of renal calculus (n)**			87.09	< 0.01
1	58 (78.4)	16 (21.6)		
> 1	52 (20.3)	204 (79.7)		

Degree of hydronephrosis: the anteroposterior diameter of the renal pelvis is measured using CT and divided into normal (0–4 mm), mild (5–9 mm), moderate (10–15 mm), and severe (> 15 mm).

Calculus residual: after applying various treatments, the stones failed to be removed cleanly.

Preoperative creatinine: the normal value of creatinine is 57–111 µmol/L.

^a^Choose to use the Fisher probability method.

^b^Clavien–Dindo I: pain was ruled out.

**Table 2:** Risk factors for moderate-to-severe postoperative pain in patients with PCNL.

Risk factor	B	Wald	*P*	OR	CI
Diameter of the renal calculus	0.001	0.007	0.001	6.23	2.50–15.56
Calculusresidual	0.577	2.716	0.01	1.780	0.897–3.533
Duration of surgery	0.032	24.883	< 0.01	1.033	1.020–1.046
Number of renal calculi	2.766	56.385	< 0.01	15.892	7.721–32.711

## Discussion

There is a global increase in the incidence of renal calculi in all age groups owing to changed dietary habits and global warming^11^. PCNL is the gold standard surgical treatment, and it plays an important role in managing especially large (> 2 cm) renal stones and/or staghorn renal stones. However, it is difficult to perform PCNL, which is greatly affected by the composition, size, and location of the stones. Severe cases are mainly complicated by septic shock and renal failure^12,13^. At present, there are few reports on the causes and risk factors of postoperative pain after PCNL. Haotian et al. showed that the incidence of moderate-to-severe postoperative pain after PCNL was about 60%^14^. It was revealed that our results were similar to Ahmet et al.’s findings which showed that the issue of PCNL-pain during postoperation period has not yet been completely solved^15^. In the present study, the clinical data of 331 patients were analyzed to identify risk factors and provide a reliable reference for clinicians to alleviate moderate-to-severe postoperative pain after PCNL.

In the present study, it was found that there were no significant differences in age, gender, BMI, ASA grade, degree of hydronephrosis, preoperative urine, culture, expansion method, tract diameter, preoperative analgesia, underlying disease, puncture time, number of expanded channels, and postoperative complications (Clavien–Dindo classification) between the two groups (P > 0.05). Multivariate logistic regression analysis showed that the independent risk factors for postoperative pain after PCNL included the diameter of the renal calculus, the number of renal calculi, the presence of residual calculi, and operation time.

Before surgery, all patients received plain computed tomography (CT) scan of the urinary system, accompanied by a high-resolution spatial presentation, and it possessed significant advantages in indicating the size, number, and location of calculi. Large calculi or large stone burdens may expand the surgical scope and prolong operation time, which can cause perfusion fluid imbalance and lead to the increase of internal pressure of the renal pelvis. The higher internal pressure of the renal pelvis, stone crushing after the release of bacteria and toxins associated with lavage by renal pelvis renal sinus, peripheral vascular and lymphatic vessels of the renal pelvis and renal pelvis venous reflux into the blood, thus, increase the risk of infection, and this leads to the release of cellular mediators, causing pain^16^.

Due to the high-resolution CT, stones containing calcium, phosphorus, and magnesium (positive stones) or uric acid stones (negative stones) can be displayed on CT images, which shows the shadow of stones with an increased density, and highlights a "calcification point". The greater the CT value (Hounsfield Units), the greater the stone hardness^13^. More time and greater intensity of lithotripsy are needed intraoperatively, aggravating the postoperative pain. It is noteworthy that partial or complete staghorn stones will have a longer operation time, a larger volume of blood loss, and more severe postoperative pain.

Postoperative pain after PCNL has important negative effects on patients’ postoperative rehabilitation, daily activities, quality of life, and social and economic conditions^17^. Inadequate analgesia can result in an increased morbidity, delayed or impaired ventilation, and prolonged hospitalization, which may increase the medical costs.

An effective management of postoperative pain is essential to reduce the morbidity of PCNL. There are several methods for reducing postoperative pain, including control of local anesthetic pressure during surgery, use of paravertebral block (T11-L1), application of erector spinae plane block, use of thoracic paravertebral block, application of quadratus lumborum block III, use of intercostal nerve block, opioid analgesia using intravenous patient-controlled analgesia, reduced size of nephrostomy tube, and no placement of a nephrostomy tube^15,18–26^. They can play a role after the onset of postoperative pain, at the same time different analgesic drugs have different adverse reactions^27^. Poorly managed acute pain has the potential to progress to chronic pain^28^, which remains a significant burden to the rehabilitation of patients. Thus, a clearer understanding of acute pain can help minimize the occurrence of acute-to-chronic pain transition.

The ERAS program has been applied to a variety of surgical procedures in recent years^29^. It aims at accelerating postoperative recovery, improving patient outcomes, and reducing medical costs^30^. An effective perioperative pain management is a crucial part of the ERAS program^31^. If we can predict and manage the risk factors for preoperative pain, perioperative pain may be reduced, thus accelerating patients’ recovery.

Our study has some limitations. Firstly, the pain intensity was examined using a self-reported scale, and as the threshold of tolerance varied from person to person, the results might be affected. Secondly, this was not a prospective, randomized controlled trial. We expected that there would be a certain correlation between Clavien–Dindo surgical complications and pain score, while it was not found. This may be related to the small sample size that influenced our results. Further multi-center studies with large sample size and long-term follow-up are therefore required to obtain more accurate clinical data for in-depth analysis. Thirdly, the high incidence of postoperation pain was found within three days after surgery. Our clinical observations showed that PCNL-pain occurred more frequently in the first 24 h, so we only evaluated the VAS pain score within 24 h after surgery not within three days. Last but not least, the pathophysiology of postoperative pain after PCNL was not explored.

## Conclusions

In summary, the diameter of the renal calculus, the number of renal calculi, the presence of residual calculi, and operation time were significant predictors of postoperative pain after PCNL. Early identification of the risk factors for moderate-to-severe postoperative pain after PCNL and formulation of reasonable preventive measures are essential to control the onset of pain and to improve patients’ quality of life.

## Data Availability

The raw data of this study are available from the corresponding author upon reasonable request.

## Abbreviations

PCNL: Percutaneous nephrolithotomy
PACU: Post-anesthesia care unit
VAS: Visual analog scale
PCIA: Patient-controlled intravenous analgesia
BMI: Body mass index

## References

[1] De Sio M, Manfredi C, Fusco F, Creta M, Mirone V, Arcaniolo D (2021). Recent advances in percutaneous lithotripsy techniques. Curr. Opin. Urol..

[2] Kyriazis I, Panagopoulos V, Kallidonis P, Özsoy M, Vasilas M, Liatsikos E (2015). Complications in percutaneous nephrolithotomy. World J. Urol..

[3] Dam M (2019). Transmuscular quadratus lumborum block for percutaneous nephrolithotomy reduces opioid consumption and speeds ambulation and discharge from hospital: A single centre randomised controlled trial. Br. J. Anaesth..

[4] Alsyouf M, Abourbih S, West B, Hodgson H, Baldwin DD (2018). Elevated renal pelvic pressures during percutaneous nephrolithotomy risk higher postoperative pain and longer hospital stay. J. Urol..

[5] Capodice JL, Parkhomenko E, Tran TY, Thai J, Blum KA, Chandhoke RA, Gupta M (2019). A Randomized, double-blind, sham-controlled study assessing electroacupuncture for the management of postoperative pain after percutaneous nephrolithotomy. J. Endourol..

[6] Amirhosseini M, Dehghan M, Mangolian Shahrbabaki P, Pakmanesh H (2020). Effectiveness of aromatherapy for relief of pain, nausea, and vomiting after percutaneous nephrolithotomy: A randomized controlled trial. Complement Med. Res..

[7] Shivanna N, Singh A, Laddha P (2018). The efficacy of tamsulosin as medical expulsion therapy in ureteric calculus of <8 mm size. J. Datta Meghe Inst. Med. Sci. Univ..

[8] Ugras MY, Toprak HI, Gunen H, Yucel A, Gunes A (2007). Instillation of skin, nephrostomy tract, and renal puncture site with ropivacaine decreases pain and improves ventilatory function after percutaneous nephrolithotomy. J. Endourol..

[9] Singh P (2016). Systemic inflammatory response syndrome following percutaneous nephrolithotomy: Assessment of risk factors and their impact on patient outcomes. Urol. Int..

[10] Su B, Hu W, Xiao B, Ding T, Liu Y, Li J (2022). Needle-perc-assisted endoscopic surgery for patients with complex renal stones: technique and outcomes. Urolithiasis.

[11] Romero V, Akpinar H, Assimos DG (2010). Kidney stones: A global picture of prevalence, incidence and associated risk factors. Rev. Urol..

[12] Ghani KR (2016). Percutaneous nephrolithotomy: Update, trends, and future directions. Eur. Urol..

[13] Karalar M (2016). Effects of parenchymal thickness and stone density values on percutaneous nephrolithotomy outcomes. Med. Sci. Monit..

[14] Haotian Wu, Huan Z (2018). Risk factors for moderate to severe postoperative pain in patients with percutaneous nephrolithotomy. Chin. J. Anesthesiol..

[15] Yayik AM, Ahiskalioglu A, Demirdogen SO, Ahiskalioglu EO, Alici HA, Kursad H (2020). Ultrasound-guided low thoracic paravertebral block versus peritubal infiltration for percutaneous nephrolithotomy: A prospective randomized study. Urolithiasis..

[16] Tokas T, Herrmann TRW, Skolarikos A, Nagele U (2019). Training and Research in Urological Surgery and Technology (T.R.U.S.T.)-Group: Pressure matters: Intrarenal pressures during normal and pathological conditions, and impact of increased values to renal physiology. World J. Urol..

[17] Gadzhiev NKG (2020). Complications after PCNL: Diagnosis and management. Urologiia..

[18] Chin KJ, Adhikary S, Sarwani N, Forero M (2017). The analgesic efficacy of pre-operative bilateral erector spinae plane (ESP) blocks in patients having ventral hernia repair. Anaesthesia.

[19] Prasad M (2020). Postoperative analgesic efficacy of fluoroscopy-guided erector spinae plane block after percutaneous nephrolithotomy (PCNL): A randomized controlled study. Saudi J. Anaesth..

[20] Yayik Ahmet Murat, et al. Ultrasound-guided low thoracic paravertebral block versus peritubal infiltration for percutaneous nephrolithotomy: A prospective randomized study. *Urolithiasis.* **48**, 235–244 (2020). 10.1007/s00240-018-01106-w 30564847

[21] Baldea KG (2020). Paravertebral block for percutaneous nephrolithotomy: A prospective, randomized, double-blind placebo-controlled study. World J. Urol..

[22] Korgün kmen, Kmen BM. Ultrasound-guided anterior quadratus lumborum block for postoperative pain after percutaneous nephrolithotomy: A randomized controlled trial. *Kor. J. Anesthesiol.* **73**,44–50 (2020). 10.4097/kja.19175 PMC7000288 31475507

[23] Chen T, Zhu ZQ, Du J (2021). Efficacy of intercostal nerve block for pain control after percutaneous nephrolithotomy: A systematic review and meta-analysis. Front. Surg..

[24] Shariat Moharari Reza, et al. Analgesic efficacy of nephrostomy tract infiltration of bupivacaine and ketamine after tubeless percutaneous nephrolithotomy: A prospective randomized trial. *Iran. J. Pharm. Res*. **15**, 619–26 (2016). PMC5018291 27642334

[25] Turmel N (2019). Use of a specific questionnaire and perineal electromyography to assess neuropathic pain after radical retropubic prostatectomy. Asian J. Urol..

[26] Chang CH, Wang CJ, Huang SW (2011). Totally tubeless percutaneous nephrolithotomy: A prospective randomized controlled study. Urol. Res..

[27] Feng D, Tang Y, Bai Y, Wei W, Han P (2020). The efficacy of local anesthetic infiltration around nephrostomy tract in alleviating postoperative pain after percutaneous nephrolithotomy: A network meta-analysis. Asian J. Surg..

[28] Dong S, Zhang K, Shi Y (2021). Carbenoxolone has the potential to ameliorate acute incision pain in rats. Mol. Med. Rep..

[29] Smith J, et al. Enhanced recovery after surgery (ERAS) program for lumbar spine fusion. *Perioper. Med. (Lond)*.**8**, 4 (2019). 10.1186/s13741-019-0114-2 PMC6537308 31149331

[30] Wu Y, Xue H, Zhang W, Wu Y, Yang Y, Ji H (2020). Application of enhanced recovery after surgery in total knee arthroplasty in patients with hemophilia A: A pilot study. Nurs. Open..

[31] Piccioni F (2018). Enhanced recovery pathways in thoracic surgery from Italian VATS Group: Perioperative analgesia protocols. J. Thorac. Dis..

